# Diffusion tensor tractography reveals muscle reconnection during axolotl limb regeneration

**DOI:** 10.1371/journal.pone.0173425

**Published:** 2017-03-02

**Authors:** Cheng-Han Wu, Yu-Jen Chen, Mu-Hui Wang, Ling-Ling Chiou, Wen-Yih Isaac Tseng, Hsuan-Shu Lee

**Affiliations:** 1 Institute of Biotechnology, National Taiwan University, Taipei, Taiwan; 2 Institute of Medical Device and Imaging, National Taiwan University College of Medicine, Taipei, Taiwan; 3 Liver Disease Prevention and Treatment Research Foundation, Taipei, Taiwan; 4 Department of Internal Medicine, National Taiwan University Hospital and National Taiwan University College of Medicine, Taipei, Taiwan; 5 Agricultural Biotechnology Research Center, Academia Sinica, Taipei, Taiwan; Mathematical Institute, HUNGARY

## Abstract

Axolotls have amazing ability to regenerate their lost limbs. Our previous works showed that after amputation the remnant muscle ends remained at their original location whilst sending satellite cells into the regenerating parts to develop into early muscle fibers in the late differentiation stage. The parental and the newly formed muscle fibers were not connected until very late stage. The present study used non-invasive diffusion tensor imaging (DTI) to monitor weekly axolotl upper arm muscles after amputation of their upper arms. DTI tractography showed that the regenerating muscle fibers became visible at 9-wpa (weeks post amputation), but a gap was observed between the regenerating and parental muscles. The gap was filled at 10-wpa, indicating reconnection of the fibers of both muscles. This was confirmed by histology. The DTI results indicate that 23% of the muscle fibers were reconnected at 10-wpa. In conclusion, DTI can be used to visualize axolotls’ skeletal muscles and the results of muscle reconnection were in accordance with our previous findings. This non-invasive technique will allow researchers to identify the timeframe in which muscle fiber reconnection takes place and thus enable the study of the mechanisms underlying this reconnection.

## Introduction

Axolotls are well known for their amazing abilities to regenerate lost limbs after amputation [1]. Amputation of the limbs results in the formation of blastemas in the stump ends, which contains undifferentiated cells capable of growing and developing into new limbs as they were before amputation [2].

In our previous work [3], we found that the regeneration of axolotl limb muscles after limb amputation does not progress piece by piece from the remnant muscle ends. Instead, satellite cells from the remnant muscle ends migrate into the blastemas since the early or mid-bud stage, and then further migrate in tracts into the regenerating limbs during the early and late differentiation stages. During the differentiation stages, the satellite cells in the proximal regenerating regions begin to differentiate into more mature muscle fibers. However, the remnant muscles stay at the original site. A gap between the regenerating and parental muscles can be found during late differentiation stage. At a more late stage, the regenerating and parental muscles join together to become well connected muscles. It is interesting and worth further investigation to see how the muscles can be reconnected accurately. However, defining the time point of the muscle reconnection can be challenging. One possible way is to detect and demonstrate the muscle reconnection with in vivo follow-up imaging by a non-invasive technique.

Diffusion tensor imaging (DTI) is a magnetic resonance imaging (MRI) technique that probes the diffusion phenomenon of water molecules at the microscopic level [4]. In soft tissues such as muscles that present high alignment of the underlying microstructures, the diffusion measured by DTI is anisotropic [5–8]. The diffusion exhibits the highest diffusivity along the long axis of the fibrous structures, and is restricted in the directions perpendicular to the long axis. It has been shown that the first eigenvector of the diffusion tensor derived from the DTI data is parallel with the local direction of the muscle fibers [9–11]. Based on this unique correspondence, reconstruction algorithm called diffusion tractography has been developed to link the local fiber orientation to obtain 3D pathways of the muscle structures [12,13]. It has been shown that diffusion tractography can be used to demonstrate fiber tract disruption in muscles, spinal cord, and cerebral white matter [14–17].

In the present study, the skeletal muscles in amputated axolotl upper arms were followed weekly by DTI over a 10-week programme. The images were further correlated with histological sections of the limbs cut off soon after the final DTI study. We aimed to show that DTI is an effective tool for longitudinal monitoring of muscle regeneration and determining the time point of reconnection between the regenerating and parental muscles.

## Materials and methods

### Ethics statement

This study was carried out in strict accordance with the recommendations in the Guide for the Care and Use of Laboratory Animals of the National Institutes of Health. The protocol was approved by the Institutional Animal Care and Use Committee of the National Taiwan University College of Medicine (Permit Number: 20120472). All surgery was performed under anesthesia, and all efforts were made to minimize suffering.

### Axolotl husbandry and experimental procedures

Axolotls were bred and cultivated in Laboratory Animal Center of National Taiwan University College of Medicine. They were kept at 18−20°C in a continuous-flow aquaria system with individual cages in an alternating 12-hour light/dark cycle. The water flowing into the system was ultraviolet-treated, bio-filtered, and its pH adjusted to 7.7−8.0. The conductivity of the water was 500−750 μSiemens/cm. The larvae were fed brine shrimp every day. The adult and juvenile axolotls were fed fish pellets three times a week. The leftovers were removed a few hours after feeding.

Amputation was performed on male adult axolotls (about one-year-old and 20 cm snout to vent length) through the middle of right upper arms. Protruding humeri resulting from retraction of surrounding soft tissues after amputation were trimmed to make the amputation plane flat. The surgical experiments were carried out under anesthesia with 0.1% MS-222 (Sigma-Aldrich, St. Louis, MO, USA).

### Imaging acquisition

**Fig 1:**
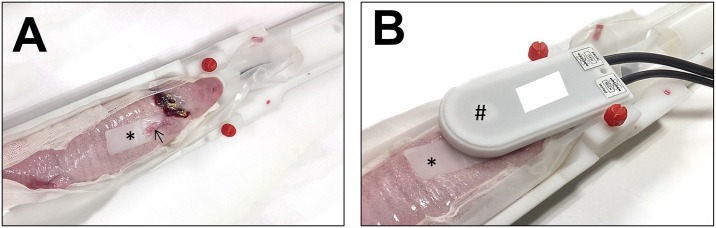
MR image acquisition. A: The anesthetized axolotl lies in left decubitus position in the trench with its right upper arm (arrow) resting in neutral position and flattened on a plastic plate (asterisk). B: A radiofrequency-receive surface coil (indicated as #) was placed on the top of the right upper arm.

The axolotl was anesthetized in a 0.1% MS-222 immersion bath for 30 min and then wrapped by 0.1% MS-222-soaked gauze and fixed in left decubitus position. The axolotl’s right upper limb was placed in a neutral position parallel to the long axis of the body (Fig 1A and 1B). Imaging was acquired on a 7T animal MRI system (BioSpin, Bruker, Houston, TX, USA) with a phased-array RF coil.

To help identify anatomic landmarks, high-resolution T2-weighted imaging of the upper limb was acquired using a fast spin-echo sequence: repetition time (TR)/echo time (TE) = 2,500/33 ms, flip angle = 180°, field-of-view (FOV) = 40 × 40 mm^2^, matrix size = 256 × 256, slice thickness = 0.63 mm, and 4 averages. DTI was acquired at the same positions as T2-weighted imaging using a pulse gradient spin-echo sequence: TR/TE = 1,500/80 ms, FOV = 40 × 40 mm^2^, matrix size = 64 × 64, slice thickness = 0.63 mm.

The diffusion encoding scheme used 12 diffusion gradient directions with diffusion sensitivity (b value) = 500 s/mm^2^, one baseline image with b value = 0, and number of averages = 6. For each axolotl, 5 to 6 long-axis slices were acquired to cover the entire thickness of the right arm.

### Fiber tracking

Reconstruction of the muscle fiber pathways was performed using streamline-based fiber tracking algorithm in DSI-Studio (http://dsi-studio.labsolver.org). The diffusion tensor was reconstructed at each voxel by applying the inverse b matrix to the diffusion-attenuated signals in 12 gradient directions [18,19]. The first eigenvector of the diffusion tensor was determined to indicate the local fiber direction within each voxel. An index called fractional anisotropy (FA) was calculated as the normalized standard deviation of the eigenvalues of the diffusion tensor, indicating the degree of directional anisotropy of diffusion. Two different approaches were adopted to examine the muscle fiber pathways [20]. In the first approach (tractography I), 10,000 seeds were placed in the right upper arm, thus including both the triceps brachii and humeroantebrachialis muscles as ROI. The parameters of this tractography were as follow: threshold of FA ≥ 0.2, turning angle = 60°, and the length of the streamlines = 5 to 20 mm (Table 1). In the second approach (tractography II), ROIs were confined to the amputation plane and the lengths of the streamlines were set in the range of 8 to 20 mm to avoid short interfering fiber tracts. Tractography II was performed to demonstrate the streamlines that indicate the fusion of the regenerating and remnant muscle fibers.

**Table 1:** Tracking parameter of two different fiber tracking approaches.

	Tractography I	Tractography II
ROI setting*	upper arm	amputation plan
*FA threshold*	0.2	0.2
*Max Angle*	60	60
*Step Size*(*mm*)	0.312	0.312
*Length Constraint*(*mm*)	5 to 20	8 to 20
*Seeds*	10,000	10,000

*Illustrated in Fig 5C.

### Preparation of tissue sections

To compare the muscle fiber tractographs with the muscle regeneration status in vivo, the limbs with desired muscle regeneration status shown on DTIs were cut through shoulders shortly after imaging, fixed in 10% neutral buffered formalin, and immersed into a decalcifying solution (Nihon Shiyaku Industries, Taipei, TW) for 24 hours. The limbs were then dehydrated in a series of gradually increased concentration of ethanol, and immersed in xylene. Finally, the limbs were embedded in paraffin for sectioning. To obtain the sections with the remnant and parental muscles continuously in the same sections, the bloc plane had to be adjusted frequently during the sectioning procedures. Consecutive 7-μm thickness sections were obtained for immunohistochemistry.

### Immunohistochemistry

The formalin-fixed, paraffin-embedded sections were deparaffinized, rehydrated, and their antigenic sites retrieved with boiling sodium citrate buffer and then blocked with phosphate-buffered saline (PBS) containing 10% fetal bovine serum at room temperature for 90 min. Subsequently, they were incubated with anti-desmin antibodies (1:200, Progen Biotechnik, Heidelberg, DE) at 4°C overnight. These sections were then rinsed in PBS containing 1% Triton X100, and their endogenous peroxidase activities were blocked in PBS containing 0.3% H_2_O_2_ at room temperature for 15 min. These sections were then incubated with horseradish peroxidase-conjugated secondary antibodies (1:200, Jackson ImmunoResearch, West Grove, PA) and developed by 3-amino-9-ethylcarbazole (DakoCytomation, Carpinteria, CA). After that, the slides were counterstained with hematoxylin and mounted with mounting medium (DakoCytomation) before microscopic examination. Images were obtained under an Olympus BX51 microscope (Olympus, Tokyo, Japan). The images were processed using Photoshop (Adobe Systems, San Jose, CA, USA) by linear adjustments.

### Statistics

Tract length and number measured by either tractography I or II in each group were presented as mean ± standard deviation. The means were compared using one-way ANOVA with post hoc Dunnett’s multiple comparison tests, and the difference between means was considered statistically significant at P< 0.05. The data of tract length and tract numbers were obtained from 3 independent axolotls in each group.

## Results

### Muscle fiber tractogram of the intact right upper arm

**Fig 2:**
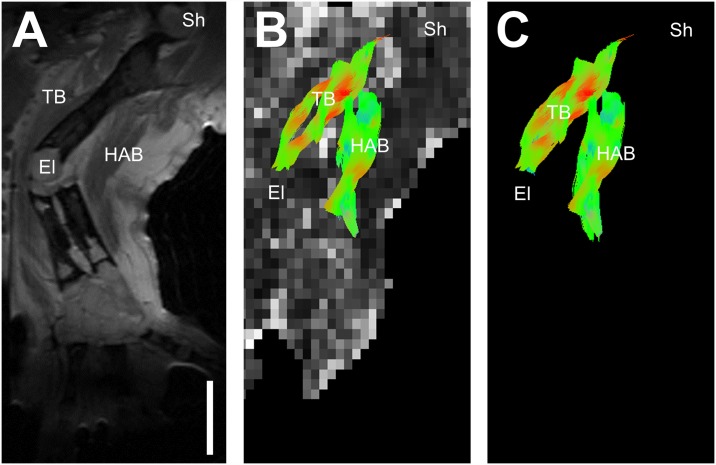
Diffusion tractography images of an adult axolotl’s right upper arm and its positioning for image acquisition. A: A T2-weighted image showing anatomical structures. B: Reconstructed fiber pathways by tractography I overlaid on the fractional anisotropy map. C: Reconstructed fiber pathways by tractography I of the entire right upper arm. Color has been assigned to indicate the direction of the local fibers; red: left-right, blue: back-front, green: up-down directions. Sh = shoulder, HAB = humeroantebrachialis, TB = triceps brachii, El = elbow. Scale bar = 5 mm.

Fig 2A–2C show images of a representative control axolotl. Fig 2A shows the T2-weighted image of the right upper arm. Fig 2B and 2C show the 3D fiber pathways reconstructed by tractography I with and without the underlying FA map, respectively. In this tractography, the region of interest (ROI) was set on right upper arm, thus including both the triceps brachii and humeroantebrachialis muscles [21], and the length of the streamlines = 5 to 20 mm. Note that the reconstructed fiber pathways showed the full length of the humeroantebrachialis and triceps brachii muscles, corresponding anatomically to those on the T2-weighted image.

### Longitudinal follow-up of the muscle fiber tractogram of the regenerating limb

**Fig 3:**
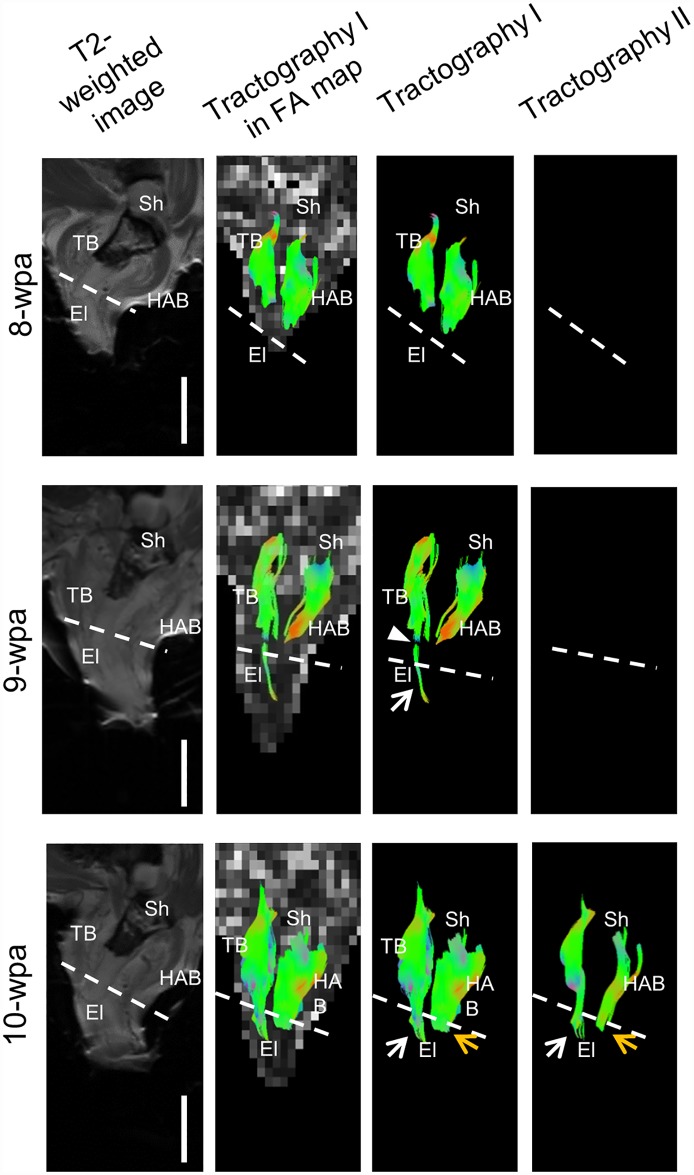
Weekly follow-up of diffusion tractography of a representative adult axolotl after amputation of the right upper arm. The images shown here were taken at 8, 9 and 10 wpa. At 8-wpa, the parental limb showed fiber pathways as reconstructed by tractography I. At 9-wpa, the regenerating part began to show triceps brachii muscle fibers (white arrow). However, the fibers were still separated from the parental fibers by a gap (white arrowhead). At 10-wpa, the regenerating part began to show fiber pathways of the humeroantebrachialis (yellow arrows). In tractography II, the fiber pathways of both triceps brachii and humeroantebrachialis crossed over the amputation plane (dash lines), implying that the muscle fibers in the regenerating part began to connect the fibers in the parental part. Sh = shoulder, HAB = humeroantebrachialis, TB = triceps brachii, El = elbow. Scale bars = 5 mm.

Fig 3 shows a series of weekly DTI images of a representative axolotl at 8, 9 and 10 weeks post-amputation (wpa). At 8-wpa, humeroantebrachialis and triceps brachii fiber pathways were seen in the parental limb but not in the regenerating limb. The images before 8-wpa were similar to those at 8-wpa and are not presented here. At 9-wpa, the fiber pathways of the triceps brachii (white arrows) reconstructed by tractography I began to appear from the regenerating limb and showed a gap separating from the parental muscle fibers (white arrowhead). At this time, there were no humeroantebrachialis fibers in the regenerating limb. At 10-wpa, the gap was filled, indicating the reconnection of the muscle fibers from the regenerating part to the parental part. Tractography II was included here. Its ROI was confined to the amputation plane and the length of the streamline was set in the range of 8 to 20 mm to avoid short interfering fiber tracts. This tractography showed very few or none of the streamlines at 9-wpa and before. However, some fiber pathways of both triceps brachii and humeroantebrachialis began to be reconstructed by this tractography at 10-wpa, further suggesting the reconnection of some regenerating and parental muscle fibers at this time point.

### Consistency of regenerating muscles between histology and DTI

**Fig 4:**
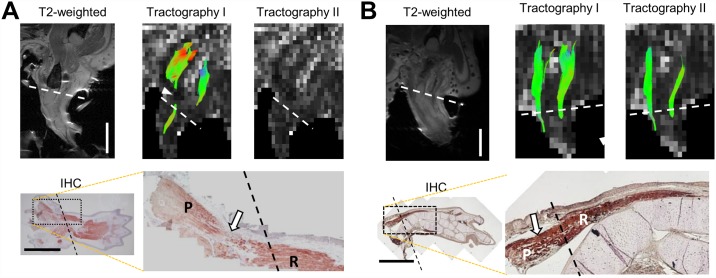
Tractography and immunohistochemistry. A: Tractography and immunohistochemistry at 9-wpa. B: Tractography and immunohistochemistry at 10-wpa. The regenerating right upper arms were amputated and embedded for sectioning soon after image acquisition. The white arrowhead in A (tractography I) indicates a gap between the parental and regenerating triceps brachii. The DTIs at 9-wpa and 10-wpa are similar to those shown in Fig 2. Note that the pathways of the regenerating humeroantebrachialis could not be seen at 9-wpa in tractography I as triceps brachii could (also see Fig 2). Desmin immunohistochemistry of the whole forelimb in A shows an expected gap (indicated by an open arrow) between the parental and regenerating humeroantebrachialis. In B, the open arrow indicates the filling of the gap in triceps brachii. P = parental muscle part. R = regenerating muscle part. Dash lines indicate amputation planes. Scale bars = 5 mm.

Given that the gap between the regenerating and parental limbs is filled on the tractogram at 10-wpa, we correlated DTI results at 9-wpa and 10-wpa with desmin immunohistochemistry (IHC) in the regenerating limbs (Fig 4). The tractograms at the 9-wpa (Fig 4A) and 10-wpa (Fig 4B) showed similar distributions of muscle fiber pathways as those in Fig 3. The right arm was re-amputated soon after image acquisition and embedded in blocs that were sectioned serially to reveal the status between regenerating and parental muscles. Because the regenerating and parental muscles of the triceps brachii or humeroantebrachialis could not always be sectioned simultaneously and longitudinally on a single slice, we could only present a humeroantebrachialis muscle at 9-wpa (Fig 4A) and a triceps brachii muscle at 10-wpa (Fig 4B). At 9-wpa, an obvious gap in the humeroantebrachialis can be observed (open arrow in IHC of Fig 4A). At 10-wpa, however, the regenerating and parental triceps brachii muscle appears to be continuous without discernable gap (open arrow in IHC of Fig 4B).

### Comparisons of the tract length and tract number between 9-wpa and 10-wpa

**Fig 5:**
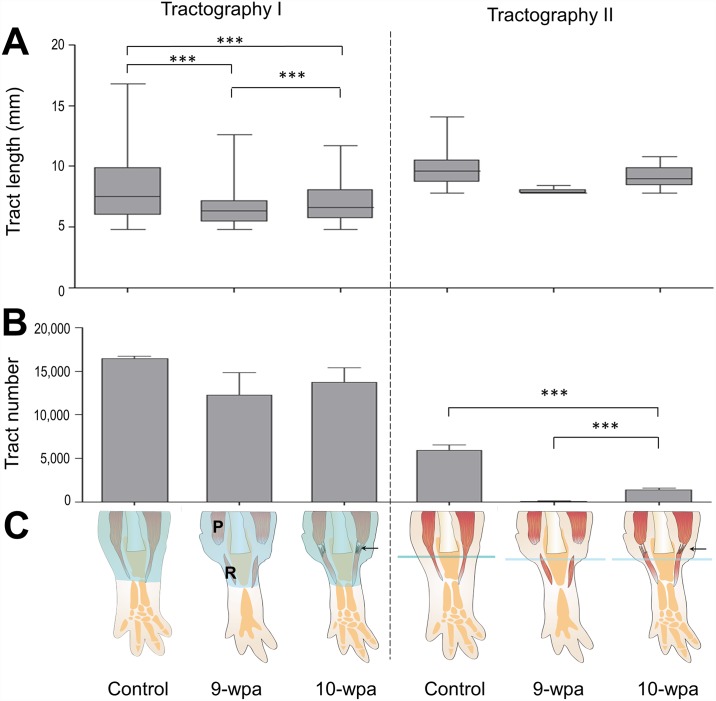
Tract lengths and tract numbers in controls and at 9-wpa, 10-wpa. A: Box plots show the distribution of the tract length (mm) in each group containing 3 individual animals. The medians in control, 9-wpa and 10-wpa by tractography I are 7.5, 6.3, and 6.6; and by tractography II are 9.8, 7.8, and 9.0, respectively. B: Bar charts show the means and standard deviation in each group respectively including 3 individual axolotls. C: Schematic illustrations of the respective ROIs by tractography I and tractography II (light blue area) and the supposed muscle fiber connections (arrows) between parental (P) and regenerating (R) muscles at 10-wpa. One-way ANOVA with post hoc Dunnett’s multiple comparison tests was used to compare the means between groups in A and B. ** P< 0.01; *** P<0.001.

By tractography I, the tract length at 9-wpa (5.5±0.8 mm) and at 10-wpa (6.7±0.3 mm) were shorter than that in control axolotls (9.7±0.7 mm; Fig 5A). The ROI in tractography I was placed at the whole upper arm (light blue shaded area in Fig 5C). Therefore, the tract length in controls should indicate the original length of the humeroantebrachialis and triceps brachii in the right upper arm. At 9-wpa and 10-wpa, most of the parental and regenerating muscle fibers were still disconnected, and so the fiber pathways reconstructed by tractography I were theoretically shorter than the original ones. Interestingly, we found that the remnant muscle fibers were retracted from the amputation plane, possibly due to muscle recoil, and remained unchanged throughout the whole regenerating period [3]. In tractography II, a linear ROI of 0.15625 mm in width was placed in the amputation plane (light blue line in Fig 5C). During the late regeneration stage, the muscle fibers that pass through the amputation plane should belong to the regenerating muscle [3]. In tractography II, only fibers longer than 8 mm (by definition, 8 to 20 mm) could be reconstructed. A minimal number of such fibers could be reconstructed at 9-wpa (79±70). However, significantly more fiber pathways (1,394±345) were recorded at 10-wpa. The length of these stream lines at 10-wpa was 9.3±0.4 mm, similar to the length of control original fibers (10.1±0.6 mm), indicating that these reconstructed muscle fibers have been elongated to the length of control fibers and approximately twice the length at 9-wpa (5.5±0.8 mm) in tractography I. It is less likely that these regenerating muscle fibers had grown and extended into the muscle origins in the shoulder in this short period of time. More likely, the regenerating muscle fibers have connected with the opposing parental fibers to become continuous fibers that could be reconstructed as a single streamline by tractography II. At 10-wpa, the tract number in tractography II was much lower than that in tractography I (1,394±345 vs. 13,762±2,893). This may be explained by the fact that the former only counted the connected fibers longer than 8 mm, while the latter counted both the connected and unconnected fibers longer than 5 mm. The stream lines in tractography II were much longer than those in tractography I (9.3±0.4 mm vs. 6.7±0.3 mm). In tractography II, the tract number at 10-wpa (1,394±345) was 23% of that found in control upper arm (5,943±1,061), suggesting 23% of the muscle fibers at 10-wpa have been reconnected.

## Discussion

The capability of the DTI method to delineate muscle microstructure had been confirmed previously [8]. However, to the best of our knowledge, this is the first DTI study to assess muscle regeneration. The aim of the present study is to show the feasibility of DTI to monitor muscle growth in axolotls after amputation, and to provide in vivo evidence to support our previous observations that the reconnection between regenerating and parental muscles occurs during late differentiation stage.

The first contribution of this study is to demonstrate that DTI is a viable technique to visualize axolotls’ limb in vivo. Muscular tissue has relatively short T2 which leads to poor signal to noise ratio (SNR) on diffusion-weighted images. To increase SNR, we used minimum TE and a surface coil with appropriate diameter. Although the use of 3D Fourier imaging can increase SNR, the scan time exceeds two hours‒ keeping axolotls under anesthesia for such a long time can be lethal. Therefore, we adopted a 2D fast spin-echo diffusion sequence. To avoid artifacts due to motion-induced phase errors, the axolotls’ limbs were immobilized tightly during the scanning. The limb immobilization proved effective because there was no motion-induced artifact in our data. The 2D fast spin-echo diffusion sequence was used to scan the axolotls’ right upper arm for 1.5 hours to obtain DTI with isotropic resolution of 0.63 mm. Pathways of the muscle fibers were reconstructed three dimensionally to provide direct visualization of the humeroantebrachialis and triceps brachii muscles (Fig 2).

Using DTI, we successfully imaged regeneration of axolotls’ limbs in vivo. In this study, DTI was used to monitor axolotls’ muscle fibers every week after limb amputation (Fig 3). Neither tractography I nor II showed fiber pathways in the regenerating muscles at time points before 9-wpa. At 9-wpa, a few fiber pathways began to appear in the regenerating triceps brachii in tractography I, but not in tractography II. At this time point, some of the regenerating triceps brachii may be longer than 5 mm which could be detected by tractography I, but below 8 mm which could not be detected by tractography II. The regenerating triceps brachii were still separated from the parental triceps brachii by a gap. In contrast, the pathways of the regenerating humeroantebrachialis could not be visualized at this time, probably because the regenerating humeroantebrachialis were shorter than 5 mm. This explanation can be supported by the fact that, at 10-wpa, the fiber pathways of the regenerating humeroantebrachialis (yellow arrows in Fig 3) below amputation plane were shorter than that of triceps brachii (white arrows in Fig 3). This phenomenon can be similarly seen in Fig 4B.

Dramatic changes were seen at 10-wpa. The gap at 9-wpa was filled in tractography I, implying that the regenerating muscles contacted the parental parts. Moreover, fiber pathways from shoulder to elbow began to appear in tractography II, indicating that these fibers were longer than 8 mm and extending from muscle origin to insertion points. It is less likely that, within a period of 7 day-interval, these fibers can elongate from the original parental or regenerating muscle and then extend beyond the amputation plane to reach the insertion or origin point, respectively. It is more plausible that the reconnection between parental and regenerating muscle fibers began to happen in this period of time.

Fiber pathways reconstructed by tractography II were apparently fewer than those by tractography I at 10-wpa, (Figs 3 and 4B), and revealed lower tract numbers (1,394±345 vs. 13,762±2,893 in Fig 5B). This indicated that only parts of the muscle fibers were reconnected at this time. This could be supported by the shorter tract length in tractography I than in tractography II at 10-wpa (Fig 5A). Comparing the tract numbers in tractography II between control (5,943±1,061) and 10-wpa (1,394±345), it can be estimated that around 23% (1,394/5,943) of the muscle fibers have been reconnected at 10-wpa. It is likely that the process of fiber reconnection continues beyond this time point.

The immunohistochemistry of the regenerating limbs at 9-wpa and 10-wpa confirmed the gap found by DTI at 9-wpa, which was filled by 10-wpa (Fig 4). However, this histological image cannot provide evidence of the reconnection between proximal and distal muscle fibers. DTI tractography shows its superiority in this aspect by providing information of individual reconnected muscle fibers.

This technique will allow researchers to know the time point when muscle fiber reconnection takes place in vivo and to study the cellular and molecular mechanisms of the process of muscle fiber reconnection in accurate proximity. For example, muscle tissues harvested during the reconnecting period as revealed by DTI can be examined under an electron microscope to elucidate how the muscle fiber ends are fused together, or subjected to transcriptome analysis for identification of the potential guidance factors for this reconnection.
